# Sennetsu Neorickettsiosis, Spotted Fever Group, and Typhus Group Rickettsioses in Three Provinces in Thailand

**DOI:** 10.4269/ajtmh.15-0752

**Published:** 2016-07-06

**Authors:** Saithip Bhengsri, Henry C. Baggett, Sophie Edouard, Scott F. Dowell, Gregory A. Dasch, Tami L. Fisk, Didier Raoult, Philippe Parola

**Affiliations:** ^1^ Thailand Ministry of Public Health–U.S. Centers for Disease Control and Prevention Collaboration, Nonthaburi, Thailand.; ^2^ Division of Global Health Protection, Centers for Disease Control and Prevention, Atlanta, Georgia.; ^3^ University Hospital Institute for Infectious Diseases and Tropical Medicine, National Reference Centre for Rickettsioses, Aix-Marseille University, Marseille, France.; ^4^ Bill & Melinda Gates Foundation, Seattle, Washington.; ^5^ Division of Vector-Borne Diseases, Centers for Disease Control and Prevention, Atlanta, Georgia.; ^6^ Division of Infectious Disease, Emory University, Atlanta, Georgia.

## Abstract

We estimated the seroprevalence and determined the frequency of acute infections with *Neorickettsia sennetsu*, spotted fever group rickettsiae, *Rickettsia typhi*, and *Orientia tsutsugamushi* among 2,225 febrile patients presenting to community hospitals in three rural Thailand provinces during 2002–2005. The seroprevalence was 0.2% for sennetsu neorickettsiosis (SN), 0.8% for spotted fever group (SFG) rickettsiae, 4.2% for murine typhus (MT), and 4.2% for scrub typhus (ST). The frequency of acute infections was 0.1% for SN, 0.6% for SFG, 2.2% for MT, and 1.5% for ST. Additional studies to confirm the distribution of these pathogens and to identify animal reservoirs and transmission cycles are needed to understand the risk of infection.

## Introduction

Rickettsioses are infectious diseases caused by obligate intracellular bacteria of the order Rickettsiales. The three groups of diseases include 1) diseases due to bacteria of the genus *Rickettsia*, including the spotted fever group (SFG) and the typhus group; 2) scrub typhus (ST) due to *Orientia tsutsugamushi*; and 3) ehrlichioses and anaplasmosis due to bacteria within the family Anaplasmataceae.^1^ In southeast Asia, rickettsial diseases have been found to be the second most commonly reported cause of non-malarial febrile illness after dengue infection.^2^ SFG rickettsioses are transmitted by arthropod vectors, including ticks, fleas, and mites.^1^,^3^,^4^ Clinical presentations can range from mild to life threatening, varying by species and region, with case fatality rates as high as 85%.^5^ SFG *Rickettsia* were initially found in ticks collected from rats (*Rattus rattus*)^6^ in one province in Thailand and identified as *R. honei*.^6^ The first three cases of serologically confirmed SFG cases in humans were detected from patients in Chiang Mai Province, Thailand.^7^ Subsequent reports identified 11 cases of SFG rickettsioses. Two cases from Chiang Rai Province were identified by polymerase chain reaction (PCR) as *R. felis*.^8^ Eight cases from Kanchanaburi Province were identified by immunofluorescent assay (IFA), western blot, and cross-adsorption as one case *R. felis*, two cases *R. conorii*, and five cases *R. helvetica*.^9^ The last case from Bangkok had titer positive for *R. honei*.^10^

ST is transmitted to humans by the bite of larval trombiculid mites, commonly known as chiggers.^11^ Murine typhus (MT) is caused by *R. typhi*, which is present in rats and mice and is known to be transmitted to humans by rat fleas.^12^ ST and MT are important causes of febrile illness in southeast Asia.^13^ The initial clinical characteristics of ST and MT can vary and include fever, cough, headache, nausea, vomiting, hepatosplenomegaly, rash, and gastrointestinal complaints.^14^–^17^ MT generally can cause moderate to severe illness; case fatality rates ranging from 1% to 4%.^16^ On the contrary, without proper treatment, patients with ST can develop severe complications with case fatality rates as high as 25%.^18^

Sennetsu neorickettsiosis (SN), caused by *Neorickettsia sennetsu* (formerly *Rickettsia sennetsu* or *Ehrlichia sennetsu*), was first described in Japan in 1954.^19^ The only published report in Thailand found that 30 (3.7%) of 812 febrile patients had positive antibody titers to *N. sennetsu*.^20^ A recent study in Laos identified and confirmed *N. sennetsu* in four (0.2%) of 1,637 febrile patients by PCR.^21^ Clinical characteristics of SN include fever, chills, myalgia, headache, hepatosplenomegaly, weakness, anorexia, lymphadenopathy, and mononucleosis.^11^,^20^ Transmission is believed to occur through consumption of raw fish (*Anabas testudineus*) contaminated with infected trematodes.^20^

Although several studies investigated the contribution of ST and MT to acute febrile illness in Thailand, large studies describing the seroprevalence and clinical characteristics of SFG and SN are limited. Clinical diagnosis and laboratory confirmation of rickettsial diseases can be challenging due to cross-reactivity or unavailability or cost of the diagnostic antigens, especially for SFG and SN, which may lead to under-recognition of these pathogens. We assessed the seroprevalence as a measure of past infection in the population and frequency of acute infection of SN, SFG, MT, and ST among patients with acute febrile illness in Thailand.

## MATERIALS AND METHODS

### Setting.

**Figure 1.**
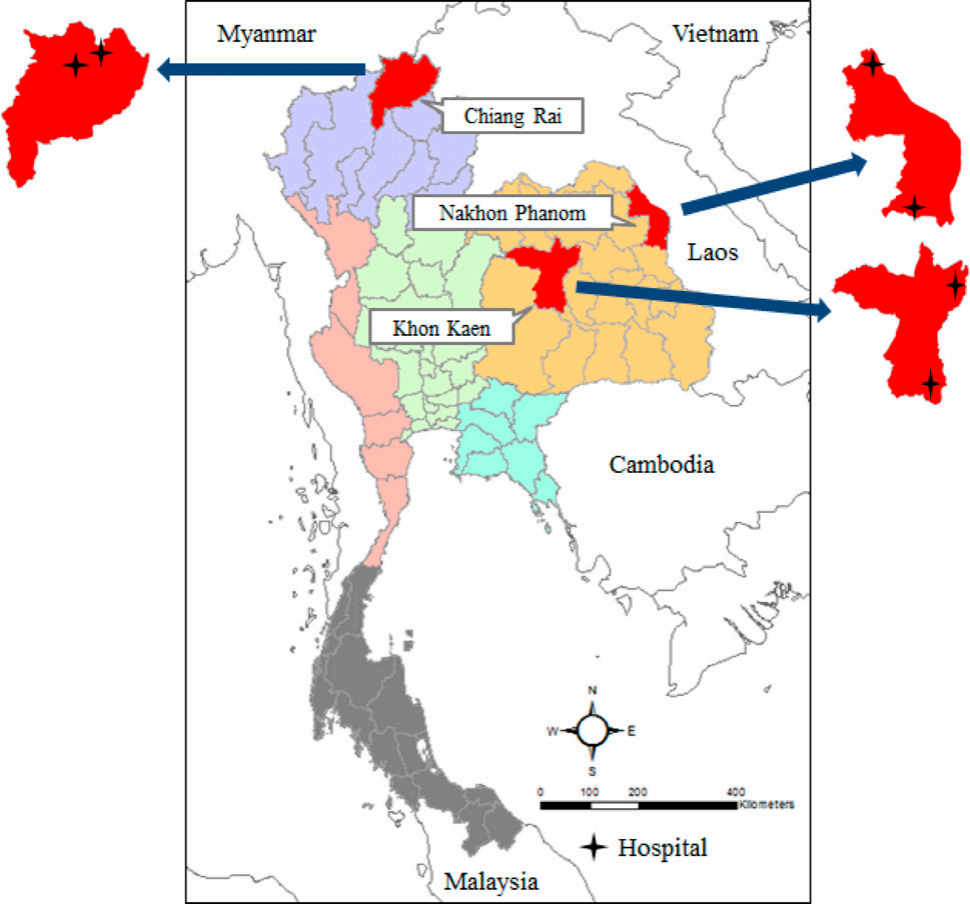
Location of study sites: two hospitals each in Chiang Rai, Khon Kaen, and Nakhon Phanom provinces, Thailand, 2002–2005.

We tested stored specimens from a prospective study of undifferentiated acute febrile illness conducted from 2002 to 2005. Enrollment occurred in two community hospitals each in Chiang Rai (northern Thailand) during 2002–2005, Khon Kaen (northeastern Thailand) during 2002–2004, and Nakhon Phanom (northeastern Thailand) during 2004–2005 (Figure 1

). These facilities ranged from 60 to 90 beds and served as the first point of care for patients requiring hospital evaluation.

### Study population.

Research staff enrolled patients aged ≥ 7 years presenting to inpatient and outpatient departments with a history of fever for less than 2 weeks and a documented temperature > 38°C at the time of evaluation. Patients were excluded if they had an apparent focal infection (e.g., streptococcal pharyngitis, meningitis, and urinary tract infection) or a specific viral infection that could be diagnosed clinically (e.g., mumps, croup, varicella, parvovirus, measles, and rubella), had received an immunization in the preceding 48 hours, had received blood products in the previous 6 months, or required immediate transfer to a larger regional hospital.

### Data and specimen collection.

Research nurses performed physical examinations and conducted interviews to collect demographic and exposure information using standard questionnaires. Potential exposures included animal or arthropod contact and outdoor activities during the 2 weeks before illness. Research nurses collected blood at enrollment and at the convalescent visit 3–5 weeks later. Serum aliquots were stored at −20°C before being transported on dry ice to the Centers for Disease Control and Prevention (CDC) laboratory in Bangkok. Sera were stored at −70°C until tested.

### Laboratory testing and interpretation.

Frozen sera were sent on dry ice to the French National Reference Center for Rickettsiosis in 2013 where immunofluorescent assays (IFA) were performed to detect anti-rickettsial antibodies as previously described.^9^ Each serum was tested for antibodies to a panel of six antigens including *N. sennetsu*, *R. honei*, *R. felis*, *R. typhi*, and *O. tsutsugamushi* (Gilliam and Kawasaki strains). All sera were screened at a dilution of 1:50 and 1:100, and those positive at 1:100 were tested by IFA to determine antibody titers. The IFA was considered positive if IgG titers were ≥ 128 and/or IgM titers were ≥ 64 in either acute or convalescent sera.^9^,^22^ For patients with serologic cross-reactions with identical titers for different antigens, we considered that the serology was positive for a given antigen if the antibody titers were greater by two dilutions compared with other antigens tested. Patients seropositive for more than one antigen at identical titers were reported as positive for both antigens; when titers to one antigen were greater by 2 dilutions, serology was considered positive for the antigen with higher titers. A seroconversion including a rise in IgG or IgM antibody titers between acute and convalescent sera was considered as a serologically confirmed acute infection.

### Statistical analysis.

Data were double entered and validated using Epi Info version 6.04 (CDC, Atlanta, GA) and analyzed using SPSS version 20.0 (SPSS Inc., Chicago, IL). Dichotomous variables were compared by χ^2^ or Fisher's exact test. Mann–Whitney *U* test was used to compare the distribution across groups. Multivariate analysis was performed for variables associated with each pathogen at a *P* value < 0.1 in univariate analysis. Results are presented as adjusted odds ratios (aORs) and 95% confidence intervals (CIs). A *P* value < 0.05 was considered significant in all calculations.

### Ethical considerations.

Research nurses obtained written informed consent from all participants ≥ 18 years of age and from the guardians of participants aged 7–17 years. Verbal assent was given by participants < 18 years of age. The protocol was approved by an institutional review board of the U.S. Centers for Disease Control and Prevention and the Ethical Review Committee, Ministry of Public Health, Thailand.

## RESULTS

**Figure 2.**
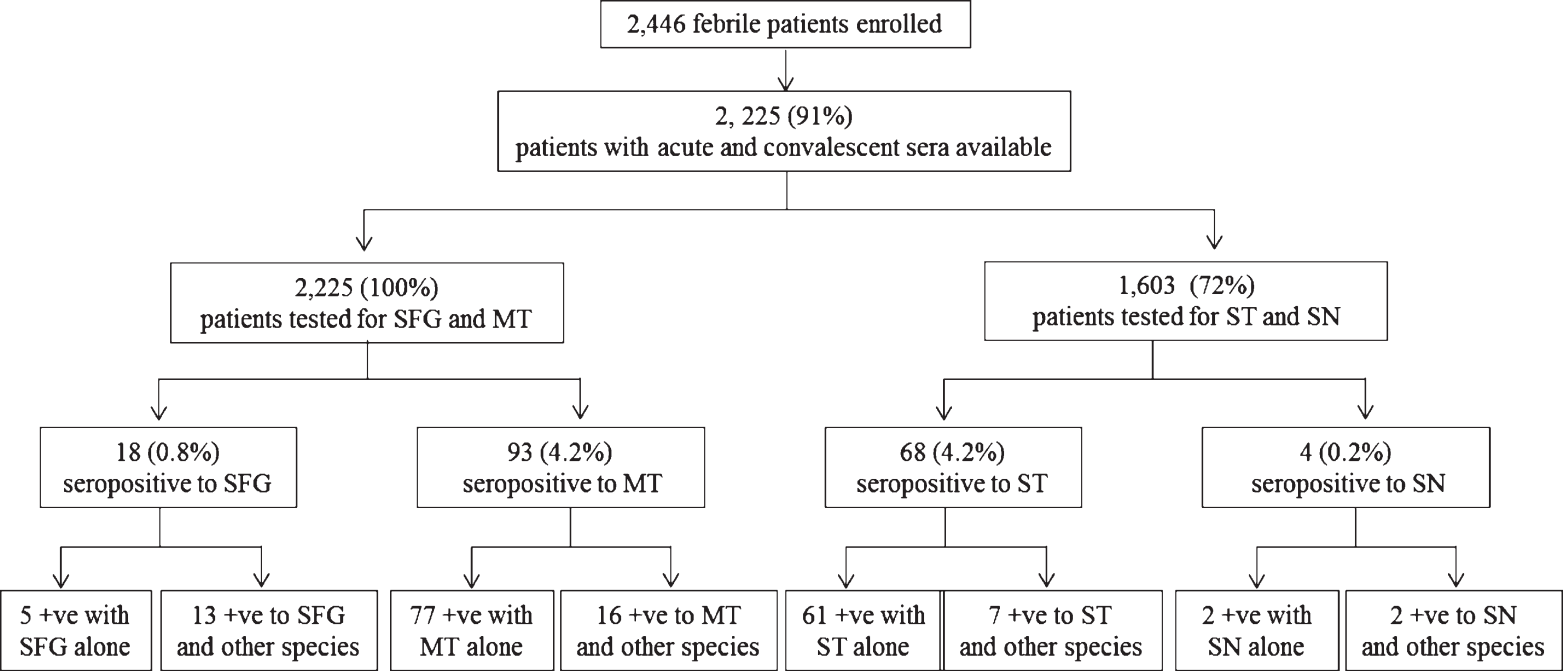
Febrile patients with positive antibody titers in acute or convalescent serum (IgG ≥ 128 or IgM ≥ 64) to spotted fever group (SFG), murine typhus (MT), scrub typhus (ST), and sennetsu neorickettsiosis (SN) in the north and northeastern Thailand, 2002–2005.

**Figure 3.**
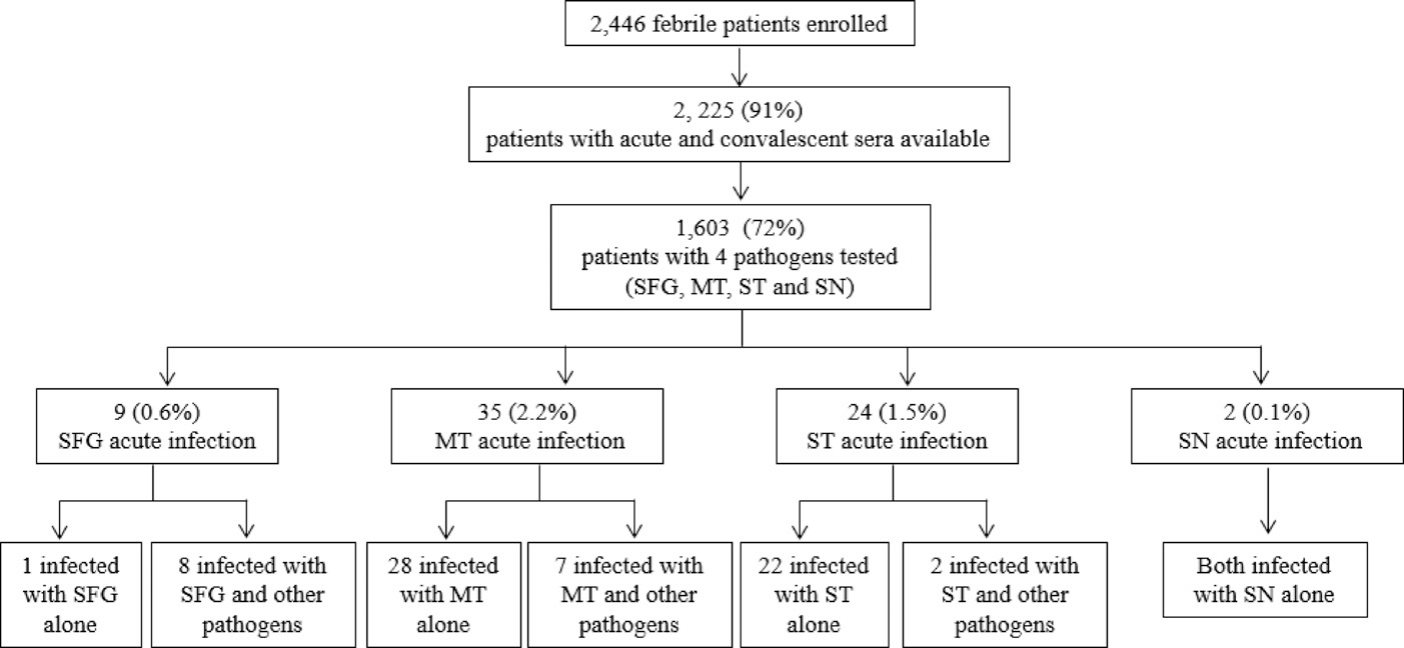
Febrile patients with serologically confirmed acute infection (a rise in IgG or IgM titers between acute and convalescent sera (i.e., seroconversion) with spotted fever group (SFG), murine typhus (MT), scrub typhus (ST), and sennetsu neorickettsiosis (SN) in the north and northeastern Thailand, 2002–2005.

During February 2002 to June 2005, we enrolled 2,446 (36%) of 6,830 eligible febrile patients, of which 63% were outpatients. The main reason for non-enrollment was refusal by the patient or guardian (59%). Of the participants, 60% were male, lived largely in rural areas (61%), and had a median age of 23 years (range = 7–89 years); 41% were students. Of the 2,446 participants, 1,105 were enrolled from hospitals in the northern region and 1,341 from the northeastern region. Ninety-one percent (2,225) of patients had acute and convalescent sera available for testing. All patients were tested for SFG and MT, while 1,603 (72%) patients were tested for SN and ST. Compared with those without serum available for SN and ST testing, participants with testing had a similar male sex distribution (52% versus 55%, *P* = 0.08) but were slightly younger (median age 25 [range 7–89] versus 31 [7–84 years], *P* < 0.01). Of 2,225 patients, 164 (7.4%) had evidence of prior infection with one or more of SFG, MT, SN, or ST (Figure 2

) and 65 (2.9%) had evidence of acute infection with at least one pathogen (Figure 3

).

### Sennetsu neorickettsiosis.

#### Seroprevalence.

Of 1,603 patients tested, four (0.2%) had a positive antibody titer to SN in acute (*N* = 2) or convalescent (*N* = 2) serum, two (0.1%) had a positive titer to SN alone, and two (0.1%) had positive titers to SN and ST (Figure 2). All four patients were from northern Thailand hospitals.

#### Acute infection.

Acute SN infection was confirmed in two (0.1%) febrile patients. Both patients tested negative for other rickettsial pathogens (Table 1 ). They were enrolled from the same hospital in northern Thailand in June and July 2002: Patient 1, a 58-year-old man, vital signs at hospital presentation were body temperature 38.1°C, respiratory rate 22 breaths/minute, pulse rate 96/minute, and blood pressure 130/80 mmHg. Patient 2, a 59-year-old woman, vital signs at hospital presentation were body temperature at hospital presentation 40.1°C, respiratory rate 20 breaths/minute, pulse rate 88/minute, and blood pressure 100/50 mmHg (Table 1).

### Spotted fever group.

#### Seroprevalence.

Of 2,225 patients tested, 18 (0.8%) had a positive antibody titer to SFG in acute (*N* = 8) or convalescent (*N* = 10) sera, five (0.2%) patients had a positive titer to SFG alone, and 13 (0.6%) had a positive titer to ≥ 1 additional rickettsial pathogen (12 positive for MT and one positive for ST). Of the five cases with positive titers to SFG alone, two were positive for *R. felis*, one positive for *R. honei*, and two had positive titers to both species. These five patients had a median age of 45 (range = 14–54) years; three were male and three were from a northeastern province.

#### Acute infection.

SFG acute infection was confirmed in nine of 1,603 (0.6%) patients. One (11%) was infected with SFG alone and eight (89%) had serologic evidence of acute infection with ≥ 1 additional pathogen based on a rise in antibody titers (six with MT, one with ST, and one with both MT and ST). The patients with evidence of acute infection to SFG alone included a 45-year-old man from northern Thailand presenting with myalgia, arthralgia, headache, cough, chest pain, abdominal pain, photophobia, and vomiting. Vital signs on admission were body temperature 40.2°C, respiratory rate 28/minute, pulse rate 100/minute, and blood pressure 140/80 mmHg. He reported having contact with animals, including cats and dogs, and being bitten by insects. He had acute IgG/IgM titers to *R. felis* of 0/0 and convalescent titers of 128/0. This patient was confirmed of having an acute infection with *R. felis* as detected by PCR.^8^

### Murine typhus.

#### Seroprevalence.

Of 2,225 febrile patients tested, 93 (4.2%) had a positive antibody titer to MT in acute (*N* = 52) or convalescent (*N* = 41) sera; 77 (3.5%) patients had a positive titer to MT alone and 16 (0.7%) had positive titers to ≥ 1 additional pathogen (12 positive for SFG and four positive for ST).

#### Acute infection.

Acute MT infection was confirmed in 35 of 1,603 (2.2%) patients. Of 35 patients with MT infection, 28 (80%) were infected with MT alone and seven (20%) had evidence of infection with ≥ 1 additional pathogen based on a rise in antibody titers (six with SFG, one with SFG and ST). Patients with MT infection alone (*N* = 28) were more likely than other febrile patients (*N* = 1,451) to have elevated alanine aminotransaminase (56% versus 27%, *P* < 0.01) and aspartate aminotransferase (74% versus 49%, *P* = 0.01) (Table 2 ). After controlling for age and sex, MT infection remained significantly associated with rodent exposure within the 2 weeks before illness (74% versus 49%, aOR = 2.9; 95% CI = 1.2–7.0).

### Scrub typhus.

#### Seroprevalence.

Of 1,603 patients tested, 68 (4.2%) had a positive antibody titer to ST in acute (*N* = 48) or convalescent (*N* = 20) sera, 61 (3.8%) patients had a positive titer to ST alone, and seven (0.4%) had positive titers to ≥ 1 additional pathogen (four positive for MT, one positive for SFG, and two positive for SN).

#### Acute infection.

ST acute infection was confirmed in 24 (1.5%) patients: 22 (92%) were infected with ST alone and two (8.3%) were infected with ≥ 1 additional pathogen based on a rise in antibody titers (one with SFG, one with MT and SFG). Compared with other febrile patients (*N* = 1,451), patients with ST infection alone (*N* = 22) more frequently had rash (32% versus 11%, *P* < 0.01), elevated alanine aminotransaminase (73% versus 27%, *P* < 0.01), and elevated aspartate aminotransferase (91% versus 49%, *P* < 0.01) (Table 2). None had eschars detected by research nurses. After controlling for age and sex, ST infection was significantly associated with insect bites (aOR = 5.0; 95% CI = 2.1–11.7) and a history of cutting down trees, clearing land, or gathering wood within the 2 weeks before illness (aOR = 2.7; 95% CI = 1.1–6.7). No ST patients received an initial diagnosis of ST.

## DISCUSSION

In a prospective study of over 2,000 patients with undifferentiated febrile illness presenting to community hospitals in northern and the northeastern Thailand, we determined the frequency of acute infection with rickettsial, ST, and SN, which cumulatively accounted for 2.9% of cases. The overall SFG, MT, and ST cases were lower than that mentioned in the previous reports (5.9–18.6%) in Thailand.^23^,^24^ However, those studies used different criteria to interpret acute infection. If we applied the same criteria, our acute infection cases will be up to 10%. Our description of SN seroprevalence and acute SN infection adds to only one previous report of SN seroprevalence in Thailand.^20^ We found an SN seroprevalence of 0.2%, which was lower than the previous study (3.7%) conducted in 2001–2003 among 812 febrile patients in northeastern and southern Thailand provinces.^20^ In contrast to that study, no SN-positive patients in our study were from the northeastern provinces.

SN was described in Japan more than 60 years ago, and it is believed to be transmitted by the consumption of raw fish contaminated with infected trematodes.^20^,^21^ Our study participants were not asked about raw fish consumption, precluding assessment of this potential exposure. Pathogenic strains of SN were also identified from the wood mouse and Norway rat (*Rattus norvegicus*) in Japan.^25^ One of our patients with acute SN infection reported recent contact with rodents, similar to a recent study in Laos,^21^ but it is unknown whether rodents have a role in disease transmission. The clinical characteristics of SN patients in our study were similar to the previous study^20^,^21^ although neither had hepatosplenomegaly or lymphadenopathy noted.

In Thailand, SFG *Rickettsia* were detected in ticks^3^,^4^,^6^ and were associated with human disease.^7^–^10^ We found SFG seroprevalence (0.8%) lower than that from a previous study (8.2–33%),^26^,^27^ but those studies used different assays (indirect immunoperoxidase test and enzyme-linked immunosorbent assay). The clinical characteristics of SFG-infected patients are generally nonspecific. Previously reported cases in Thailand had fever, headache, injected conjunctivae, lymphadenopathy, and rash.^7^,^9^ Patients in our study with acute SFG infection alone had fever and headache, but had no lymphadenopathy or rash. Almost three-quarters of patients in our study with evidence of acute infection with both SFG and MT reported rodent exposure within the 2 weeks before illness. If these patients were truly coinfected, one could speculate that the infections may have occurred through a common transmission pathway.

The seroprevalence of ST (4.2%) was similar to that of a previous report among Thai soldiers from northeastern Thailand (4.1%),^28^ but that study used a lower antibody titer of ≥ 50 to define positivity. Using the same cutoff, seroprevalence in our study would be 5.6%. Clinical and laboratory characteristics that distinguished ST patients from other febrile patients included a higher prevalence of rash, lymphadenopathy, thrombocytopenia, elevated liver enzymes, and a lower prevalence of leukocytosis.^15^,^29^ The presence of an eschar (the dark crusted ulcer at the site of chigger bites) is often considered a hallmark of ST^30^ but based on clinician reports and study nurse examinations; no patients with confirmed ST in our study had an eschar observed. Studies on children ≤ 15 years with ST have found a range of eschar prevalence from 7.2% (5/69) to 70% (21/30),^15^,^31^ while one previous report noted a lack of eschar in 15 ST patients.^32^ Although a thorough examination for eschar is warranted in patients with undifferentiated illness in ST-endemic areas, disease cannot be ruled out by the lack of eschar. It is possible that our research nurses failed to detect eschars in some patients.

MT has a worldwide distribution and is more prevalent in southeast Asia.^33^ The seroprevalence of MT in our study (4.2%) was lower than in a previous study (8.0%) in suburban Bangkok,^33^ which used the indirect immunoperoxidase assay and defined seropositivity as antibody titers > 50. Using the same cutoff for positivity in our study would have given a seroprevalence of 6.3%. Elevated liver enzymes were common among patients with MT, consistent with previous reports. Previous reports of MT cases also described frequent anemia, thrombocytopenia, and leukopenia^22^,^34^,^35^; in our study, anemia occurred in 29%, but thrombocytopenia in 7.1% and leucopenia in 10%. We found that rodent exposure was independently associated with MT, consistent with the fact that rat fleas are known vectors.^36^

The nonspecific clinical presentation of rickettsioses may lead to treatment delay if clinicians are not aware of the frequency of disease. We found that none of SFG cases and only one of 65 *Rickettsia* cases overall received a corresponding clinical diagnosis. SFG, ST, or MT would not respond to empiric treatment and needed to be treated with doxycycline.^29^ Underdiagnosis can also delay appropriate antimicrobial therapy, which has been associated with poor patient outcomes.^18^ The low percentage of patients with the correct diagnosis results at least partially from the lack of valid diagnostic tests that can be performed in a community hospital laboratory. A laboratory test that could be performed at the facility level could greatly improve diagnosis and treatment of febrile illness in Thailand.

Our study had several limitations. We enrolled only patients seeking care in community hospitals, patients requiring immediate transfer to a larger regional hospital were not enrolled, and only 36% of eligible febrile patients participated, potentially limiting the generalizability of our findings. The small number of serologically confirmed SFG and SN cases limited the ability to assess clinical characteristics and risk factors for these diseases. Although serology testing is the standard for diagnosing rickettsial diseases and IFA is generally the serological method of choice, serologic assays are known to cross-react, especially assays for SFG with assays for other rickettsial species.^9^,^37^ IFA results suggesting coinfection with > 1 rickettsiosis should be interpreted in light of this known cross-reactivity. Although direct detection methods, such as PCR, were not used, we did use conservative serologic cutoffs to define seropositivity. Western blot and cross-adsorption assays are recommended to be used to distinguish *Rickettsia* species,^1^ but we lacked sufficient sera for testing.

Our findings on MT and ST place renewed importance on measures to prevent these diseases. Clinicians should maintain a level of suspicion for these important zoonotic diseases and consider appropriate antibiotic therapy (e.g., doxycycline) for patients with clinically consistent illnesses without delay or waiting for laboratory diagnostic results. Additional investigations are warranted to understand the epidemiology of SFG and SN in Thailand, including animal reservoirs and transmission cycles. Laboratory capacity should be strengthened to support diagnosis of rickettsial diseases to refine prevalence estimates and improve data to inform control strategies.

## SUMMARY

This study determined the frequency of acute infection with SN, SFG and typhus group rickettsioses, as well as the seroprevalence of these pathogens among febrile patients in Thailand.

## Figures and Tables

**Table 1:** Characteristics of febrile patients with serologically confirmed acute infection with *Neorickettsia sennetsu*, northern Thailand, 2002–2005

Characteristics	Patient 1	Patient 2
Age (year)	58	59
Gender	Male	Female
Occupation	Day laborer	Messenger
Initial symptoms	Fever 6 days, headache, fatigue, cough, chest pain, myalgia, arthralgia, diarrhea, vomiting, and dyspepsia	Fever 5 days, fatigue, cough, chest pain, myalgia, arthralgia, and gastrointestinal symptoms
Initial diagnosis	Pharyngitis	Enteric fever
Hospitalization	No	Yes
Leukocyte count, cells/mm^3^	5,200	7,200
Platelet count, cells/mm^3^	187,000	274,000
Hemoglobin, g/dL	13.4	13.1
Blood urea nitrogen, mg/dL	32	14.1
Creatinine, mg/dL	0.7	0.8
Alanine aminotransferase, IU/dL	24.6	12.0
Aspartate aminotransferase, IU/dL	23.8	0.5
Bilirubin, mg/dL	0.5	0.3
Alkaline phosphatase, IU/dL	198	45
History of animal contact within 2 weeks before illness onset	Mosquito bites	Cats, dogs, rodents, and mosquito bites
History of eating raw fish	No information	No information
Acute titers, IgG/IgM	0/0	0/0
Convalescent titers, IgG/IgM	256/0	256/0

**Table 2:** Characteristics of MT and ST patients compared with other febrile patients with negative serology testing to any rickettsial species (non-cases), Thailand, 2002–2005

Characteristics	MT		ST		Non-cases
	*N* = 28 (%)	*P* value*	*N* = 22 (%)	*P* value*	*N* = 1,451 (%)
Median age	34.5	0.03	24.5	0.9	28
Male sex	16 (57)	0.8	14 (67)	0.4	800 (55)
Headache	23 (82)	0.9	20 (91)	0.3	1,181 (81)
Cough	11 (39)	0.01	9 (41)	0.04	909 (63)
Myalgia	23 (82)	0.08	14 (64)	0.8	967 (67)
Arthralgia	9 (32)	0.6	9 (41)	0.7	528 (36)
Rash	4 (14)	0.6	7 (32)	< 0.01	158 (11)
Lymphadenopathy	0 (0.0)	0.04	6 (27)	0.05	188 (13)
Hepatosplenomegaly	0 (0.0)	0.6	0 (0.0)	0.7	13 (0.9)
Hospitalization	15 (54)	0.07	9 (41)	0.7	536 (37)
White blood cell > 10,000 mm^3^	6 (21)	0.2	0 (0.0)	< 0.01	469 (32)
Hemoglobin ≤ 12 g/dL	8 (29)	0.5	5 (23)	0.3	492 (34)
Platelet ≤ 149 mm^3^	5 (18)	0.8	8 (36)	0.05	285 (20)
Platelet ≤ 100 mm^3^	2 (7.1)	0.7	6 (27)	< 0.01	81 (5.6)
Blood urea nitrogen ≥ 21 mg/dL	1 (3.7)	0.2	1 (4.5)	0.3	159 (11)
Creatinine ≥ 1.5 mg/dL	0 (0.0)	0.1	1 (4.5)	0.5	125 (8.6)
Alanine aminotransaminase ≥ 36 IU/dL	15 (56)	< 0.01	16 (73)	< 0.01	392 (27)
Aspartate aminotransferase ≥ 36 IU/dL	20 (74)	0. 01	20 (91)	< 0.01	712 (49)
Bilirubin ≥ 1.3 mg/dL	4 (14)	0.2	2 (9.1)	0.9	118 (8.2)
Alkaline phosphatase ≥ 121 IU/dL	19 (68)	0.4	18 (82)	0.1	946 (65)

MT = murine typhus; ST = scrub typhus.

* *P* value for comparison between patients with corresponding rickettsial infection and other febrile patients with negative serology testing for any rickettsial species or sennetsu neorickettsiosis (non-cases).
